# MIBE acts as antagonist ligand of both estrogen receptor α and GPER in breast cancer cells

**DOI:** 10.1186/bcr3096

**Published:** 2012-01-17

**Authors:** Rosamaria Lappano, Maria Francesca Santolla, Marco Pupo, Maria Stefania Sinicropi, Anna Caruso, Camillo Rosano, Marcello Maggiolini

**Affiliations:** 1 Dipartimento Farmaco-Biologico, Università della Calabria, via Bucci, 87036 Rende, Italy; 2 Dipartimento di Scienze Farmaceutiche, Università della Calabria, via Bucci, 87036 Rende, Italy; 3 U.O.S. Biopolimeri e Proteomica, Azienda Ospedaliera Universitaria IRCCS San Martino IST - Istituto Nazionale per la Ricerca sul Cancro, Largo Benzi 10, 16132 Genova, Italy

## Abstract

**Introduction:**

The multiple biological responses to estrogens are mainly mediated by the classical estrogen receptors ERα and ERβ, which act as ligand-activated transcription factors. ERα exerts a main role in the development of breast cancer; therefore, the ER antagonist tamoxifen has been widely used although its effectiveness is limited by *de novo *and acquired resistance. Recently, GPR30/GPER, a member of the seven-transmembrane G protein-coupled receptor family, has been implicated in mediating the effects of estrogens in various normal and cancer cells. In particular, GPER triggered gene expression and proliferative responses induced by estrogens and even ER antagonists in hormone-sensitive tumor cells. Likewise, additional ER ligands showed the ability to bind to GPER eliciting promiscuous and, in some cases, opposite actions through the two receptors. We synthesized a novel compound (ethyl 3-[5-(2-ethoxycarbonyl-1-methylvinyloxy)-1-methyl-1H-indol-3-yl]but-2-enoate), referred to as MIBE, and investigated its properties elicited through ERα and GPER in breast cancer cells.

**Methods:**

Molecular modeling, binding experiments and functional assays were performed in order to evaluate the biological action exerted by MIBE through ERα and GPER in MCF7 and SkBr3 breast cancer cells.

**Results:**

MIBE displayed the ability to act as an antagonist ligand for ERα and GPER as it elicited inhibitory effects on gene transcription and growth effects by binding to both receptors in breast cancer cells. Moreover, GPER was required for epidermal growth factor receptor (EGFR) and ERK activation by EGF as ascertained by using MIBE and performing gene silencing experiments.

**Conclusions:**

Our findings provide novel insights on the functional cross-talk between GPER and EGFR signaling. Furthermore, the exclusive antagonistic activity exerted by MIBE on ERα and GPER could represent an innovative pharmacological approach targeting breast carcinomas which express one or both receptors at the beginning and/or during tumor progression. Hence, the simultaneous inhibition of both ERα and GPER may guarantee major therapeutic benefits in respect to the use of a selective estrogen receptor antagonist.

## Introduction

Estrogens regulate many aspects of human physiology and influence diverse pathological processes, including the development of hormone-dependent tumors [1]. The biological actions of estrogens are mainly mediated by the estrogen receptor (ER)α and ERβ, which belong to the nuclear receptor superfamily [1]. Acting as ligand-activated transcription factors, ERs regulate gene expression by binding to responsive elements (ERE) located within the promoter region of estrogen target genes [1]. In addition, gene regulation can occur in response to estrogens through plasma membrane receptors, such as growth factor receptors or G protein-coupled receptors, and by protein kinase signaling cascades [2].

Prolonged exposure to estrogens represents a major risk factor for the progression of breast cancer [3], which expresses elevated levels of ERα in approximately 70% of cases [4]. Consequently, ERα antagonists like tamoxifen and raloxifene are currently used as frontline pharmacological interventions in ERα-positive breast cancer in order to inhibit the mitogenic stimulation of estrogens [5]. Although there is general concordance between ERα expression and responsiveness to ER-targeted agents, as indicated by a greater five-year disease-free survival for ERα-positive patients receiving tamoxifen, one in four patients does not respond to treatment from the onset and in most patients tamoxifen produces agonist effects after a few years [6].

In order to further characterize the molecular mechanisms involved in the action of estrogens, recent studies have demonstrated that the G protein-coupled receptor, named GPR30/GPER, mediates rapid biological responses to estrogens in diverse normal, as well as transformed, cell types [7]. The potential role of GPER in cancer was supported by numerous investigations performed in different tumor cells, including breast [8-10], endometrial [11], ovarian [12], thyroid [13], prostate [14] and testicular germ cells [15]. In accordance with these findings, GPER has been associated with aggressive features of breast cancer [16], high-grade endometrial tumors [17] and poor prognosis in ovarian cancer [18]. Since its identification to date, the transduction signaling and gene expression profile triggered by GPER have been extensively characterized. The early discovery [8] of a transmembrane receptor able to mediate estrogen responsiveness in ER-negative breast cancer cells was then confirmed by several reports by which GPER was considered as a genuine ER [10,19]. Indeed, a whole series of intracellular events, such as the rapid phosphorylation of mitogen-activated protein kinases (MAPK) ERK1/2, the activation of PI3-kinase (PI3K) and phospholipase C (PLC), the increase in cAMP concentrations and the intracellular calcium mobilization, was shown to follow GPER activation by both estrogens and anti-estrogens [20]. In particular, it was demonstrated that GPER-dependent ERK activation occurs via the transactivation of the epidermal growth factor receptor (EGFR) through matrix metalloproteinase activity and integrin α5β1, which trigger the extracellular release of heparan-bound epidermal growth factor (HB-EGF) [8,21]. Interestingly, a physical and functional cross-talk between GPER and EGFR contributes to the intricate signaling network involved in the stimulation of hormone-sensitive tumors [22,23].

The rapid responses to estrogenic signals mediated by GPER regulate a typical gene signature, as revealed in previous studies, including a microarray analysis [7,24]. Of note, GPER target genes were shown to contribute to the proliferation and migration in diverse cancer cell types [9,11-13,22,24,25] as well as in cancer associated fibroblasts (CAFs) [26].

GPER exhibits many of the expected characteristics of an estrogen receptor, including the capability to bind to estrogens, phyto- and xenoestrogens and even the ER antagonists 4-hydroxytamoxifen (OHT) and fulvestrant (ICI 182 780) [10,19,27,28]. Surprisingly, unlike the antagonistic properties displayed by these anti-estrogens with respect to the classical ERs, both compounds act as GPER agonists [8,11,19,24]. Conversely, the well known ER agonist estriol exerts inhibitory effects on GPER-mediated signaling [28], confirming the potential opposite functions elicited by estrogenic/anti-estrogenic agents through each type of estrogen receptor. In addition to the selective GPER agonist G-1 [29], GPER ligands showing antagonistic properties have been identified [30,31]. Recently, a GPER antagonist showed at high concentrations limited binding properties and stimulatory activity on ER-mediated transcription [30]. The use of these compounds has greatly advanced our understanding of the role of GPER in numerous biological systems as well as in cancer.

On the basis of the aforementioned findings, GPER may be considered as an additional therapeutic target in estrogen-sensitive tumors, such as breast cancer. In this regard, the opposite functional activity elicited by anti-estrogens through the classical ERs and GPER as stated above, could represent a therapeutic concern toward the pharmacological inhibition of all types of estrogen receptor.

**Figure 1:**
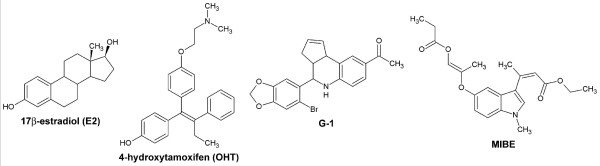
**Chemical structures of compounds used**. 17beta-estradiol (E2), 4-hydroxytamoxifen (OHT), G-1 and ethyl 3-[5-(2-ethoxycarbonyl-1-methylvinyloxy)-1-methyl-1H-indol-3-yl]but-2-enoate (MIBE).

We discovered a novel compound, ethyl 3-[5-(2-ethoxycarbonyl-1-methylvinyloxy)-1-methyl-1H-indol-3-yl]but-2-enoate (referred to as MIBE) (Figure 1), which displays the unique property to bind to and inhibit GPER- and ERα-mediated signaling in breast cancer cells. The antagonistic action exerted by MIBE on both estrogen receptor types could represent a novel, promising tool for a more comprehensive pharmacological approach to estrogen-dependent tumors such as breast cancer.

## Materials and methods

### Molecular modelling and docking simulations

For docking simulations we used as targets the crystallographic coordinates of ERα in complex with E2 (closed-conformation) as well as with OHT (open conformation) and a GPER molecular model built by homology as described elsewhere (PDB code 1G50; PDB code 3ERT) [28,32,33]. Docking studies were performed by GOLD 5.0.1 (the Cambridge Crystallographic Data Center, UK), a program using a genetic algorithm useful to investigate the full range of ligand conformational flexibility and a partial protein side chain flexibility. As active sites of ERα, we identified those atoms that are within 20 Å distance from each atom of the ligand experimental position. Regarding GPER, we identified the O atom of Phe 208 as the protein active site centre on the basis of our previous docking simulations [28]. In this case, the active site atoms were considered those located within 20 Å from the centre. For each structure, 10 docking solutions were generated allowing an early termination of the process, if the respective RMSDs of the three highest ranked docking solutions were within 1.5 Å of each other. The default GOLD settings were used for running the simulations. ERα protein side chains Met342, Glu353, Trp383, Met388, Arg394, Phe404, His524 and Leu525 were considered as flexible, while in the GPER model the residues Tyr123, Gln138, Phe206, Phe208, Glu275, Phe278 and His282 were defined flexible side chains allowing their free rotation. The molecular structures of the ligands screened *in silico *were built and energy minimized with the programs Insight II and Discover3 (Biosym/MSI, San Diego, CA, USA). All the figures were drawn with the program Chimera (UCSF, San Francisco, CA, USA) [34].

### Chemistry

5-Hydroxy-1-methylindole was allowed to react with an excess of ethyl acetoacetate using Indium(III) chloride as a catalyst. The derivative ethyl 3-[5-(2-ethoxycarbonyl-1-methylvinyloxy)-1-methyl-1H-indol-3-yl]but-2-enoate (MIBE) was obtained in good yield [35,36]. Melting points were determined on a Kofler melting point apparatus. IR spectra were taken with a Perkin Elmer BX FT-IR (Corporate Headquarters, Waltham, Massachusetts, USA). Mass spectra were taken on a JEOL JMS GCMate spectrometer at ionising potential of 70 eV (EI). ^1^H-NMR (400 MHz) was recorded on a JEOL Lambda 400 Spectrometer (JEOL Ltd., Tokyo, Japan). Chemical shifts are expressed in parts per million downfield from tetramethylsilane as an internal standard. Thin layer chromatography (TLC) was performed on silica gel 60F-264 (Merck, Frankfurt, Germany). Commercial reagents were purchased from Aldrich Chemical (Milan, Italy), Acros Organics (Carlo Erba Reagenti S.p.A., Rodano, Milan, Italy) and Alfa Aesar (Karlsruhe, Germany). Unless otherwise stated, all commercial reagents were used without further purification.

Procedure for the preparation of MIBE was as follows. Indium (III) chloride (10 mol%) was added under nitrogen to a mixture of 5-hydroxy-1-methyl-1H-indole and ethyl acetoacetate. The reaction mixture was heated under reflux for two hours, and then it was left to cool to room temperature. Ice water was added and then the reaction mixture was extracted by ethyl acetate. The organic layers were collected and washed with brine, dried over MgSO_4 _and evaporated under reduced pressure. The solid residue was washed with Et_2_O, to give the pure compound MIBE a pink solid, yield of 65%, mp = 180°C; IR (KBr): 3412, 2984, 1705, 1622, 1473, 1373, 1168, 1088, 1027, 805 cm^-1^. ^1^H-NMR (d_6_-DMSO) δ 8.94 (s, 1H, Ar); 7.32 (d, 1H, Ar, J_7,6 _= 8.8 Hz); 6.87 (s, 1H, Ar); 7.32 (d, 1H, Ar, J_6,7 _= 8.8 Hz); 6.04-6.01 (m, 2H, C = CH); 4.11-4.09 (q, 2H, CH_2_); 3.90-3.88 (q, 2H, CH_2_); 3.76 (s, 3H, NCH_3_); 1.42 (s, 6H, C-CH_3_); 1.24-1.20 (t, 3H, CH_3_); 0.95-0.92 (t, 3H, CH_3_). MS (EI) m/z: 371 (M^+^, 14).

### Reagents

17β-estradiol (E2), 4-hydroxytamoxifen (OHT) and 5α-dihydrotestosterone (DHT) were purchased from Sigma-Aldrich (Milan, Italy). G-1 (1-[4-(-6-bromobenzol [1,3]diodo-5-yl)-3a,4,5,9b-tetrahidro3H5 cyclopenta[c]quinolin-8yl]-ethanone) was bought from Calbiochem (Merck KGaA, Frankfurt, Germany). All compounds were solubilized in ethanol, except G-1 and MIBE which were dissolved in dimethyl sulfoxide (DMSO).

### Cell culture

MCF7 breast cancer cells and human embryonal kidney Hek293 cells were maintained in DMEM with phenol red supplemented with 10% FBS. SkBr3 breast cancer cells were maintained in RPMI 1640 without phenol red supplemented with 10% FBS. All cell lines to be processed for immunoblot and RT-PCR assays were switched to medium without serum and phenol red the day before treatments.

The experiments performed in this study do not require Institute Ethics Board approval, because only commercially available cell lines were used.

### Plasmids

Firefly luciferase reporter plasmids used were ERE-luc for ERα [37], ARE-luc for the Androgen Receptor (AR) [38] and GK1 [37] for the Gal4 fusion proteins Gal-ERα and Gal-ERβ, which were expressed from plasmids GAL93.ER(G) and GAL93.ERβ, respectively, as previously described [37]. The full length AR expression plasmid (AR) was previously described [39]. As the internal transfection control, we co-transfected the plasmid pRL-TK (Promega, Milan, Italy) that expresses *Renilla *Luciferase. Short hairpin RNA construct against human GPER (shGPR30/shGPER) and the unrelated shRNA control construct were previously described [22].

### Transfection, Luciferase assays and gene silencing experiments

Cells were plated into 24-well plates with 500 μl of regular growth medium/well the day before transfection. Cell medium was replaced with medium supplemented with 1% charcoal-stripped (CS) FBS lacking phenol red and serum on the day of transfection, which was performed using the Fugene 6 Reagent as recommended by the manufacturer (Roche Diagnostics, Milan, Italy) with a mixture containing 0.5 μg of reporter plasmid, 2 ng of pRL-TK, 0.1 μg of effector plasmid and 0.1 μg of full length AR expression plasmid where applicable. After 6 h, the medium was replaced again with serum-free medium lacking phenol red and supplemented with 1% CS-FBS, treatments were added at this point and cells were incubated for an additional 18 h. Luciferase activity was then measured using the Dual Luciferase Kit (Promega, Milan, Italy) according to the manufacturer's recommendations. Firefly luciferase activity was normalized to the internal transfection control provided by the *Renilla *luciferase activity. The normalized relative light unit values obtained from cells treated with vehicle were set as one-fold induction upon which the activity induced by treatments was calculated.

For the gene silencing experiments, cells were plated into 10-cm dishes, maintained in serum-free medium for 24 h and then transfected for an additional 48 h before treatments using Fugene 6 (according to the manufacturer's recommendations) and control vector (shRNA) or shGPER.

### Ligand binding assays

In ligand binding assay for ERα, the ability of MIBE to compete with [3H]E2 was evaluated and compared with that of E2. Two picomoles of purified recombinant human ERα protein purchased from PanVera, Invitrogen S.r.l. (Milan, Italy), each in 20 mM HEPES, pH 7.4, 1.5 mM EDTA, 0.5 mM dithiothreitol, and 10% (v/v) glycerol, was incubated with 1 nM [2,4,6,7-3H]E2 (89 Ci/mmol; Ge Healthcare, Milan, Italy) and increasing concentrations of nonlabeled E2 or MIBE for two hours at 37°C in a humidified atmosphere of 95% air/5% CO2. Bound and free radioligands were separated on Sephadex G-25 PD-10 columns. The amount of receptor-bound [3H]E2 was determined by liquid scintillation counting.

In ligand binding assay for GPER, SkBr3 cells were grown in 10-cm cell culture dishes, washed two times and incubated with 1 nM [2,4,6,7-3H]E2 (89 Ci/mmol; Ge Healthcare, Milan, Italy) in the presence or absence of an increasing concentration of nonlabeled competitors (E2, G-1, OHT and MIBE). Then, cells were incubated for two hours at 37°C and washed three times with ice-cold PBS; the radioactivity collected by 100% ethanol extraction was measured by liquid scintillation counting. Competitor binding was expressed as a percentage of maximal specific binding. Each point is the mean of three observations.

### Reverse transcription and real-time PCR

Gene expression was evaluated by real-time PCR as we previously described [37]. For Cyclin D1, IRS-1, PR, pS2, c-fos, CTGF, Cyr61, EGR1, and the ribosomal protein 18S, which was used as a control gene to obtain normalized values, the primers were: 5'-GTCTGTGCATTTCTGGTTGCA-3' (Cyclin D1 forward) and 5'-GCTGGAAACATGCCGGTTA-3' (Cyclin D1 reverse); 5'-GCCCGTGTTACTGTTCATTCAG-3' (IRS-1 forward) and 5'-AATAACGGACACTGCACAACAGTCT-3' (IRS-1 reverse); 5'-GAGTTGTGAGAGCACTGGATGCT-3' (PR forward) and 5'-CAACTGTATGTCTTGACCTGGTGAA-3' (PR reverse); 5'-GCCCCCCGTGAAAGAC-3' (pS2 forward) and 5'-CGTCGAAACAGCAGCCCTTA-3' (pS2 reverse); 5'-CGAGCCCTTTGATGACTTCCT-3' (c-fos forward), 5'-GGAGCGGGCTGTCTCAGA-3' (c-fos reverse); 5'-ACCTGTGGGATGGGCATCT-3' (CTGF forward), 5'-CAGGCGGCTCTGCTTCTCTA-3' (CTGF reverse); 5'-GAGTGGGTCTGTGACGAGGAT-3' (Cyr61 forward) and 5'-GGTTGTATAGGATGCGAGGCT-3' (Cyr61 reverse); 5'-GCCTGCGACATCTGTGGAA-3' (EGR1 forward), 5'-CGCAAGTGGATCTTGGTATGC-3' (EGR1 reverse); and 5'- GGCGTCCCCCAACTTCTTA -3' (18S forward) and 5'- GGGCATCACAGACCTGTTATT -3' (18S reverse), respectively.

### Western blotting

Cells were grown in 10-cm dishes, exposed to ligands, and then lysed in 500 μL of 50 mmol/L NaCl, 1.5 mmol/L MgCl2, 1 mmol/L EGTA, 10% glycerol, 1% Triton X-100, 1% sodium dodecyl sulfate (SDS), and a mixture of protease inhibitors containing 1 mmol/L aprotinin, 20 mmol/L phenylmethylsulfonyl fluoride and 200 mmol/L sodium orthovanadate. Protein concentration was determined using Bradford reagent according to the manufacturer's recommendations (Sigma-Aldrich, Milan, Italy). Equal amounts of whole protein extract were resolved on a 10% SDS-polyacrylamide gel, transferred to a nitrocellulose membrane (GE Healthcare, Milan, Italy), probed overnight at 4°C with antibodies against Cyclin D1 (M-20), IRS-1 (A-19), c-fos (H-125), CTGF (L-20), GPER (N-15), pEGFR Tyr 1173 (sc-12351), β-actin (C-2), phosphorylated ERK1/2 (E-4) and ERK2 (C-14) (all purchased from Santa Cruz Biotechnology, DBA, Milan, Italy), and then revealed using the ECL™ Western Blotting Analysis System (GE Healthcare, Milan, Italy).

### Proliferation assay

For quantitative proliferation assay, cells (1 × 10^5^) were seeded in 24-well plates in regular growth medium. Cells were washed once they had attached and then were incubated in medium containing 2.5% charcoal-stripped FBS with the indicated treatments; medium was renewed every two days (with treatments) before counting, using the Countess Automated Cell Counter, as recommended by the manufacturer's protocol (Invitrogen S.r.l., Milan, Italy).

### Statistical analysis

Statistical analysis was done using ANOVA followed by Newman-Keuls' testing to determine differences in means. *P *< 0.05 was considered as statistically significant.

## Results

### Molecular modeling and binding assays demonstrate that MIBE is a ligand of both ERα and GPER

**Figure 2:**
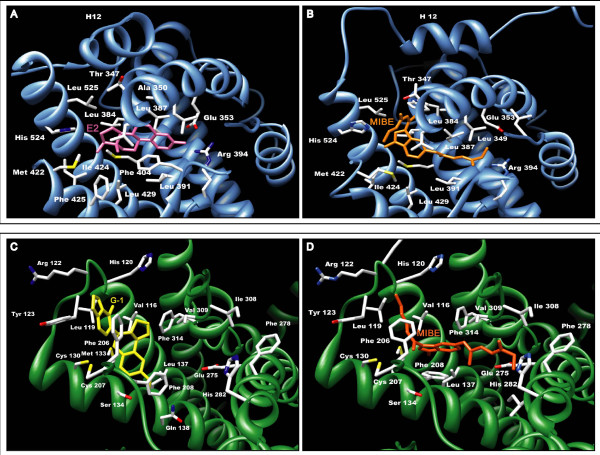
**GPER and ERalpha docking simulations**. (a-b) The three-dimensional model of ERalpha is schematically reported as a light blue ribbon cartoon; residues involved in ligand binding are drawn as sticks. (a) The binding modes of E2 (pink sticks) to ERalpha in the "closed conformation" is shown. (b) The MIBE moiety (orange sticks) is drawn in its favorable conformation bound to ERalpha (open conformation), with the helix 12 displaced with respect to the position exhibited in the ERalpha-E2 complex. (c-d) The GPER model is reported as green ribbon and residues involved in ligand binding are drawn as sticks. (c) G-1 is drawn in yellow. (d) The MIBE moiety is drawn as orange sticks.

On the basis of the results obtained in docking simulations as described in the Materials and methods section, we evaluated the affinity of MIBE for the ligand binding pockets of both ERα and GPER with respect to E2 and G-1, respectively (Figure 2). Docking E2 to the hormone binding pocket of a closed conformation of ERα (Figure 2a), we observed a binding mode similar to that reported in the experimental crystallographic complex (superposition of the solution provided by GOLD to the crystallographic structure led to a RMSD of 0.092Å) [32]. Docking MIBE to the same pocket using ERα in both the closed and open conformation, we evidenced a better affinity for the last conformation (Figure 2b) and a binding mode similar to that adopted by the ER antagonist OHT in the crystallographic structure (PDB code 3ERT) [33].

As it concerns the GPER ligand binding pocket, visual inspection showed that it lies within a deep cleft in where 10 hydrophobic residues (V116, Met133, Leu137, Phe206, Phe208, Phe 278, Ile279, Ile308, Val309 and Phe314) and 5 polar amino acids (Tyr123, Gln138, Asp210, Glu275 and His282) contribute to stabilize the ligands through Van der Waals interactions and hydrogen bonds, respectively. Using GPER as a target, docking simulations confirmed a good affinity of the protein for the agonist G-1 (Figure 2c) as previously demonstrated both *in silico *and *in vitro *[29]. Next, we docked MIBE to GPER using the same settings and parameters as for G-1. MIBE, which was positioned within the GPER binding site (Figure 2d), displayed a high affinity for GPER, even better than that exhibited by G-1. In particular, MIBE binds to GPER forming hydrogen bonds with the hydroxyl groups located on its branched arms, on one side with Y123 OH, on the other with Q215 NE2 and H282 ND1 atoms. MIBE is also stabilized in the protein binding pocket by Van der Waals interactions of its methyl groups with residues F208, I279, T305 and I308, while a π-π stacking interaction is formed by the aromatic rings of F208 and the indole ring of MIBE. Starting from the aforementioned observations, we performed diverse assays to fully evaluate the ligand binding properties and the potential agonist/antagonist activity of MIBE exerted through ERα and GPER.

**Figure 3:**
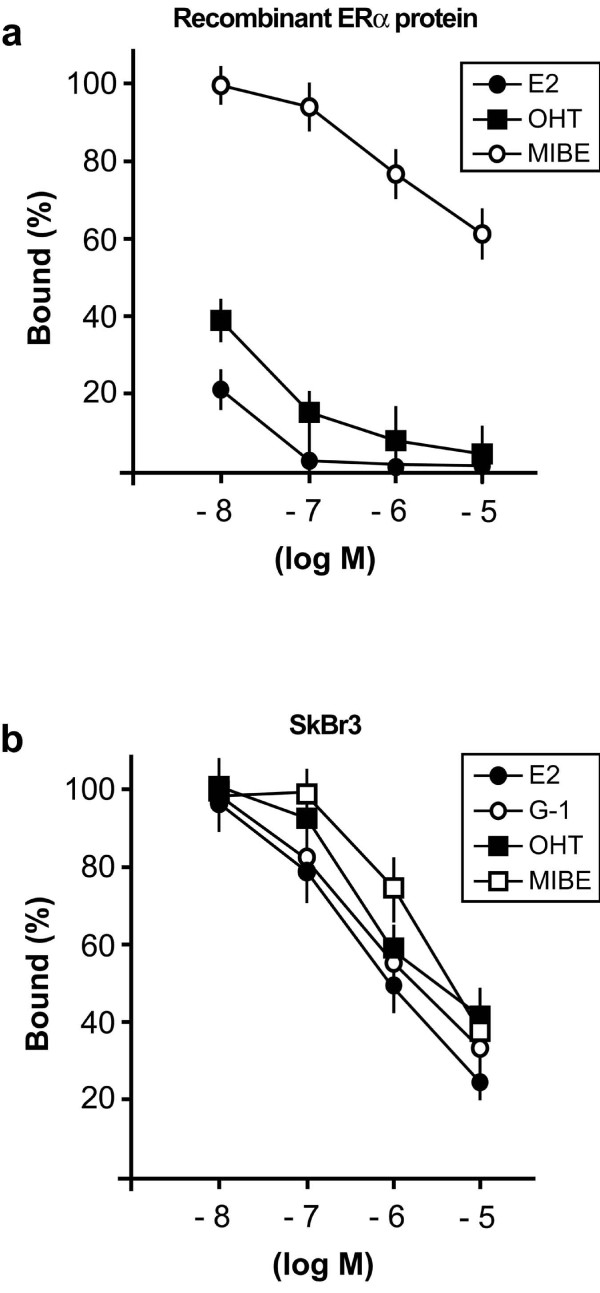
**MIBE is a ligand of GPER and ERalpha**. (a) MIBE competes with [3H]E2 for the binding to ERalpha. Competitive binding of increasing concentrations of unlabelled E2, OHT and MIBE to recombinant human ERalpha protein. Each data point represents the mean ± SD of triplicate samples of three separate experiments. (b) Ligand binding assay in SkBr3 cells. Competition curves of increasing concentration of unlabelled E2, G-1, OHT and MIBE expressed as a percentage of maximum specific [3H]E2 binding. Each data point represents the mean ± SD of three separate experiments performed in triplicate.

In order to confirm whether MIBE is a ligand of ERα, we performed competitive binding experiments by using the recombinant ERα protein. MIBE displaced the radiolabeled E2 in a dose-dependent manner (Figure 3a) indubitably demonstrating its capability to bind to ERα in a direct fashion, although with a lower binding affinity in respect to E2 and OHT as 10 μM MIBE induced approximately 40% displacement of [3H]E2. On the basis of the ability of MIBE to interact with GPER in docking simulations, we also performed ligand binding studies using radiolabeled E2 as a tracer in ER-negative but GPER-positive SkBr3 breast cancer cells, as previously reported [28]. Hence, we performed binding experiments using cold E2, MIBE, the selective GPER ligand G-1 and OHT, which has been largely reported to act as a GPER agonist [7]. Interestingly, MIBE showed the capability to displace [3H]E2 (Figure 3b) in accordance with the results obtained in docking simulations. E2, G-1 and OHT confirmed the ability to compete with [3H]E2 as previously shown [28]. Collectively, our findings demonstrate that MIBE is a ligand of both ERα and GPER.

### MIBE inhibits both ER transactivation and gene expression induced by E2

**Figure 4:**
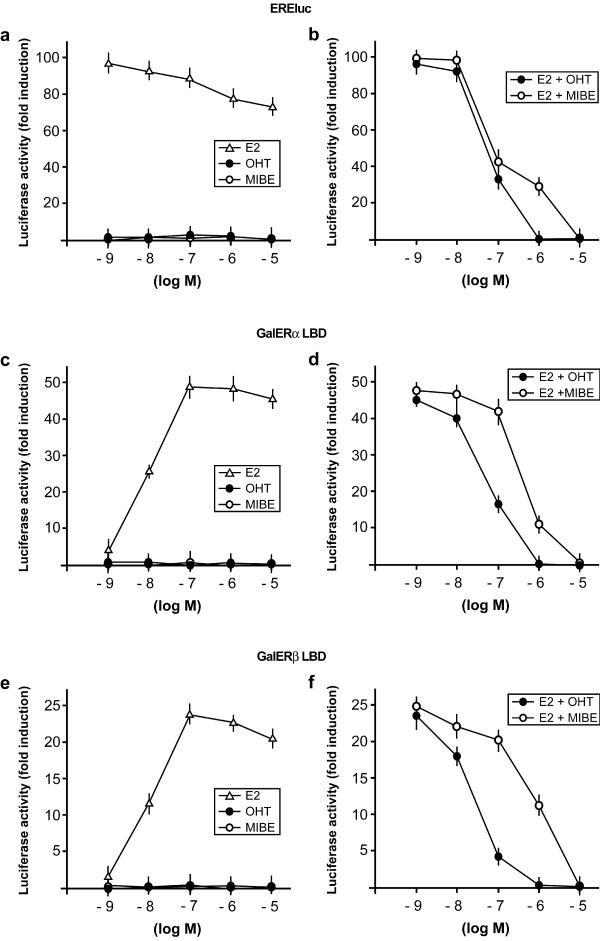
**MIBE inhibits the transactivation of ERalpha induced by E2**. (a) MCF7 cells were transfected with the ER luciferase reporter gene (EREluc) along with the internal transfection control Renilla Luciferase and treated with increasing concentrations (logarithmic scale) of E2, the ER antagonist OHT and MIBE. (b) MCF7 cells were transfected with the ER reporter gene and the internal transfection control Renilla Luciferase and treated with 10 nM E2 in combination with increasing concentration of OHT or MIBE, as indicated. (c, e) Hek293 cells were transfected with Gal4 reporter gene GK1, the Gal4 fusion proteins encoding the Ligand Binding Domain (LBD) of ERα (GalERalpha) or ERbeta (GalERbeta) and the internal transfection control Renilla Luciferase and treated with increasing concentrations (logarithmic scale) of E2, OHT and MIBE. (d, f) Hek293 cells were transfected with the Gal4 reporter gene GK1, the Gal4 fusion proteins GalERalpha or GalERbeta and the internal transfection control Renilla Luciferase and treated with 100 nM E2 in combination with increasing concentrations of OHT or MIBE, as indicated. Each data point represents the mean ± SD of three experiments performed in triplicate.

On the basis of these results, we aimed to ascertain whether MIBE could function as an agonist or antagonist for ERα and GPER. Initially, we evaluated the potential of MIBE in activating or inhibiting the ERα-mediated signaling. Hence, we transiently transfected an ER-reported gene in MCF7 breast cancer cells, which express ERα but not ERβ as judged by RT-PCR (data not shown). The reporter plasmid used carries firefly luciferase sequences under the control of an ERE upstream of the thymidine kinase promoter. As an internal transfection control, we co-transfected a plasmid expressing renilla luciferase which is enzymatically distinguishable from firefly luciferase by the strong cytomegalovirus enhancer/promoter. MIBE did not show any capability to transactivate ERα; however, it abrogated the luciferase activity induced by E2 like the ER antagonist OHT (Figure 4a, b). To confirm these data and to examine the response of ERβ, we transiently transfected the ER-negative Hek293 cells with chimeric proteins consisting of the DNA binding domain (DBD) of the yeast transcription factor Gal4 and the ligand binding domain (LBD) of ERα (GalERα) or ERβ (GalERβ), respectively. MIBE did not activate GalERα and GalERβ (Figure 4c, d), but prevented the transactivation of these chimeric proteins by E2 mimicking the inhibitory activity of OHT (Figure 4e, f). In order to evaluate whether MIBE acts through a further member of the steroid receptor superfamily as the AR, we transiently transfected the ER-negative Hek293 cells with an AR reporter gene along with the expression vector encoding AR. DHT transactivated the AR reporter gene, whereas MIBE neither activated AR nor prevented the DHT-induced activation of AR (Additional file 1). Together, these results provide evidence regarding the specific action of MIBE on ER-mediated signaling.

**Figure 5:**
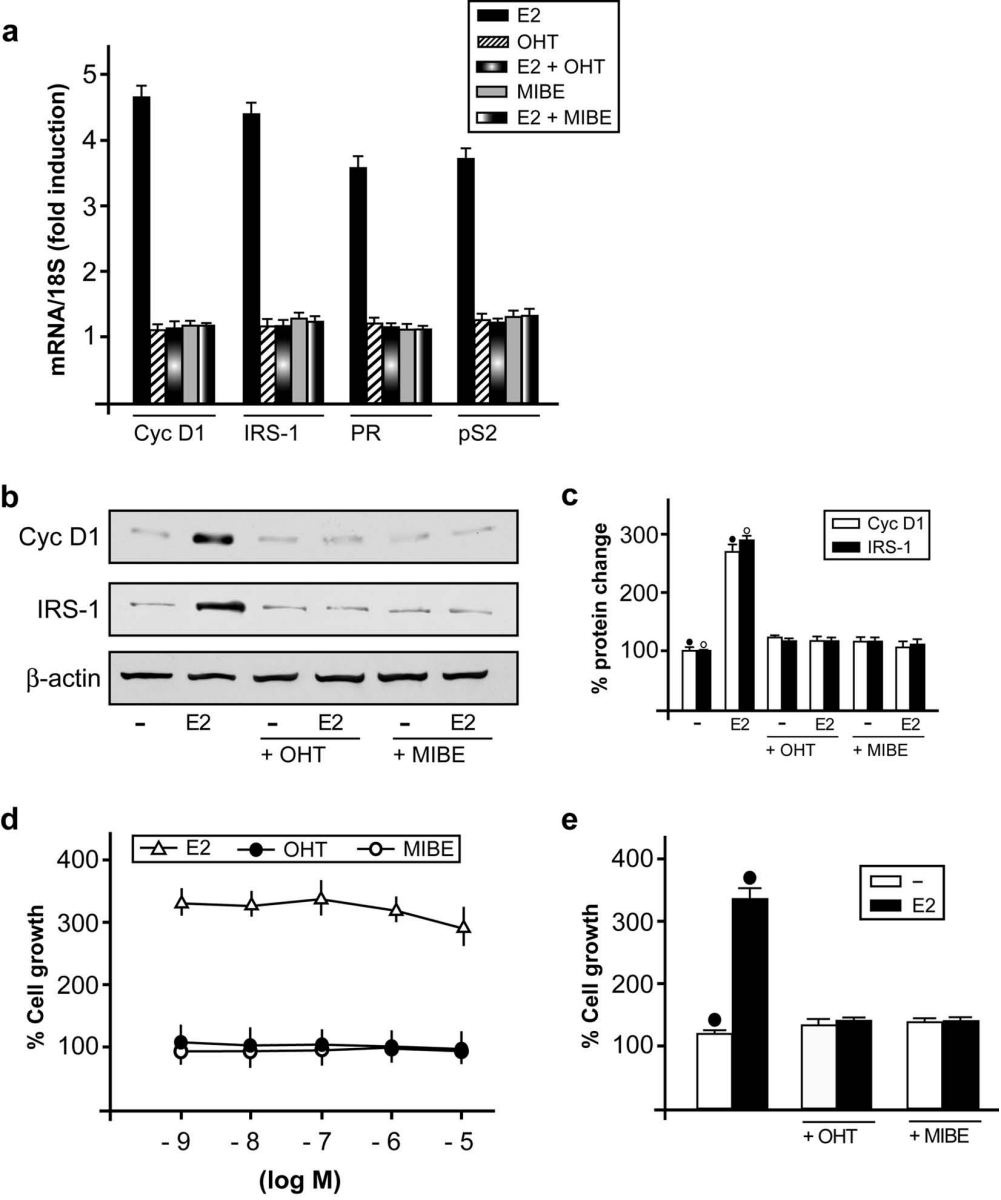
**MIBE inhibits gene expression and proliferation induced by E2 in MCF7 cells**. (a) Evaluation of mRNA expression of Cyclin D1 (Cyc D1), IRS-1, Progesterone Receptor (PR) and pS2 by real-time PCR in MCF7 cells. Cells were treated for 24 h with vehicle, 10 nM E2, 1 microM OHT and 10 microM MIBE alone or in combination, as indicated. Results obtained from experiments performed in triplicate were normalized for 18S expression and shown as fold change of RNA expression compared to cells treated with vehicle. Each data point represents the mean ± SD of three independent experiments performed in triplicate. (b) Immunoblots of protein levels of Cyclin D1 (Cyc D1) and IRS-1 from MCF7 cells. Cells were treated for 24 h with vehicle (-), 10 nM E2, 1 microM OHT and 10 microM MIBE alone or in combination, as indicated. β-actin serves as loading control. Data shown are representative of three independent experiments. (c) Densitometric analysis of three independent experiments, protein expressions are normalized to beta-actin. (•), (◦) indicate *P *< 0.05 for cells receiving vehicle versus treatments. (d) MCF7 cells were treated for five days with vehicle, increasing concentrations (logarithmic scale) of E2, OHT and MIBE and counted on Day 6. (e) Cells were treated for five days with vehicle (-), 10 nM E2, 1 microM OHT and 10 microM MIBE alone or in combination, as indicated, and then the proliferation was evaluated by cell counts on Day 6. The proliferation of cells receiving vehicle was set as 100% upon which cell growth induced by treatments was calculated. Each data point is the average ± SD of three independent experiments performed in triplicate. (•) indicates *P *< 0.05 for cells receiving vehicle (-) versus treatments.

In order to further demonstrate that MIBE acts as an ERα antagonist, we evaluated its ability to repress in MCF7 cells the mRNA expression of well known E2 target genes like pS2, Cyclin D1, PR and IRS-1. As determined by real-time PCR, the E2-dependent increase of all genes examined was prevented by MIBE as obtained using OHT (Figure 5a). Similarly, the protein expression of cyclin D1 and IRS-1 induced by E2 in MCF7 cells was inhibited by MIBE (and OHT) (Figure 5b, c).

### MIBE prevents the proliferative effects triggered by E2

Considering that the regulation of estrogen target genes connects the signaling of E2 with the proliferation of breast cancer cells [40,41], we wanted to determine the biological significance of the antagonist action elicited by MIBE through ERα. MIBE as OHT did not stimulate growth effects used alone (Figure 5d); however, both compounds abolished the proliferation of MCF7 cells induced by E2 (Figure 5e). Hence, MIBE can be considered as an ER antagonist on the basis of its full inhibitory activity elicited on ER-mediated signaling.

### MIBE prevents the GPER-mediated EGFR and ERK activation

**Figure 6:**
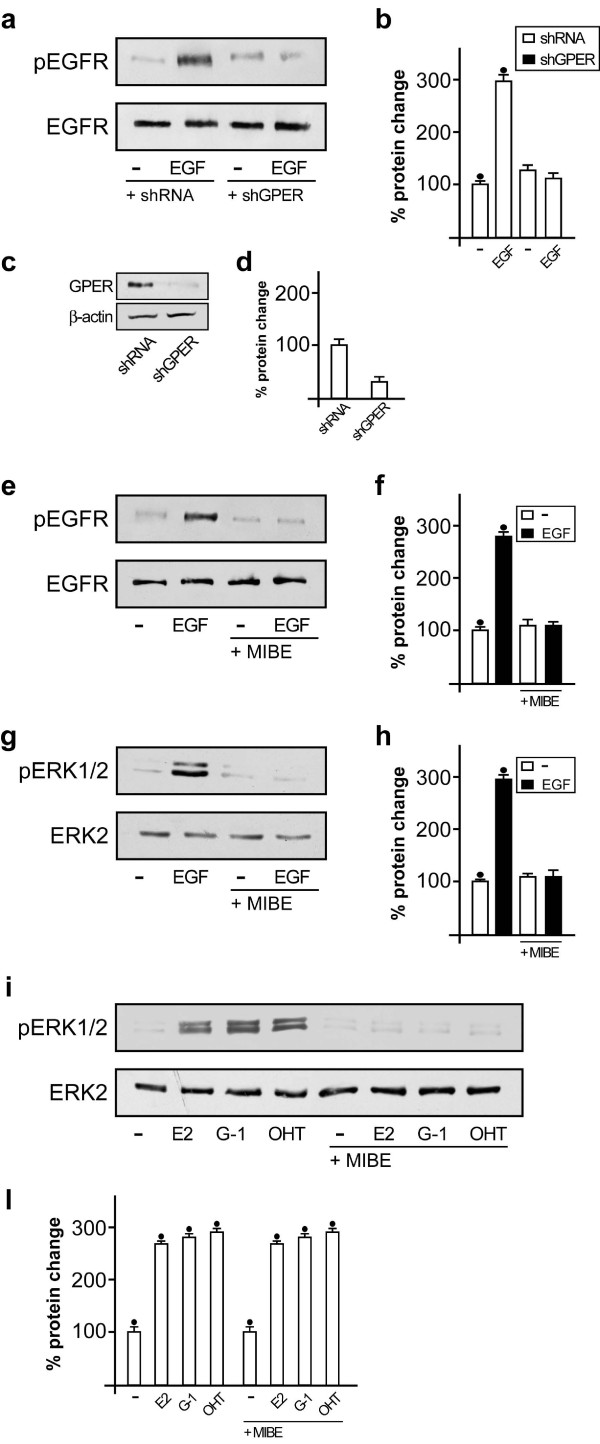
**MIBE prevents the phosphorylation of EGFR and ERK1/2**. (a) EGFR^Tyr1173 ^phosphorylation after treatment (five minutes) with vehicle (-) and 100 ng/ml EGF in SkBr3 cells transfected with shRNA or shGPER. (b) Densitometric analysis of three independent experiments, EGFR^Tyr1173 ^expressions are normalized to EGFR. (c) Efficacy of GPER silencing obtained using shGPER. (d) Densitometric analysis of three independent experiments. GPER expressions are normalized to beta-actin. (e) EGFR^Tyr1173 ^phosphorylation after treatment (five minutes) with vehicle (-) and 100 ng/ml EGF alone and in combination with 10 μM MIBE. (f) Densitometric analysis of three independent experiments. EGFR^Tyr1173 ^expressions are normalized to EGFR. (g) ERK1/2 activation in SkBr3 cells treated for five minutes with vehicle (-) or 100 ng/ml EGF alone and in combination with 10 microM MIBE. (h) Densitometric analysis of three independent experiments. ERK1/2 expressions are normalized to ERK2. (i) ERK1/2 activation in SkBr3 cells treated for 15 minutes with vehicle (-), 100 nM E2, 1 microM G-1 and 5 microM OHT alone and in combination with 10 microM MIBE. Data shown are representative of three independent experiments. (i) Densitometric analysis of three independent experiments. ERK1/2 expressions are normalized to ERK2. (•) indicates *P *< 0.05 for cells receiving vehicle versus treatments.

Having established that MIBE is an inhibitor of ERα, we aimed to determine its functional activity on the GPER-mediated transduction pathway. Previous studies have indicated that GPER activation triggers the EGFR-dependent signaling in cancer cells, even involving a functional cross-talk between these receptors [8,9,23]. Then, we sought to evaluate the role played by GPER in EGFR phosphorylation upon exposure to its cognate ligand. Notably, in SkBr3 cells the EGFR activation induced by EGF was prevented by knocking down GPER expression (Figure 6a-d) as observed in the presence of MIBE (Figure 6e, f), which further demonstrated that it acts as an inhibitor of GPER-mediated function. Accordingly, the activation of EGFR triggered by G-1 was abolished in the presence of MIBE, hence confirming its inhibitory activity on GPER-mediated signaling (Additional file 2). Corroborating the aforementioned findings, MIBE showed the capability to inhibit the ERK activation upon EGF exposure (Figure 6g, h) as well as by the GPER activators E2, G-1 and OHT (Figure 6i-l). Overall, these results suggest that MIBE acting as an inhibitor of GPER blocks the EGFR activation and the ERK phosphorylation induced by EGF and the ligands of GPER, thus preventing the functional cross-talk between GPER and EGFR.

### MIBE inhibits gene transcription and cell proliferation mediated by GPER

**Figure 7:**
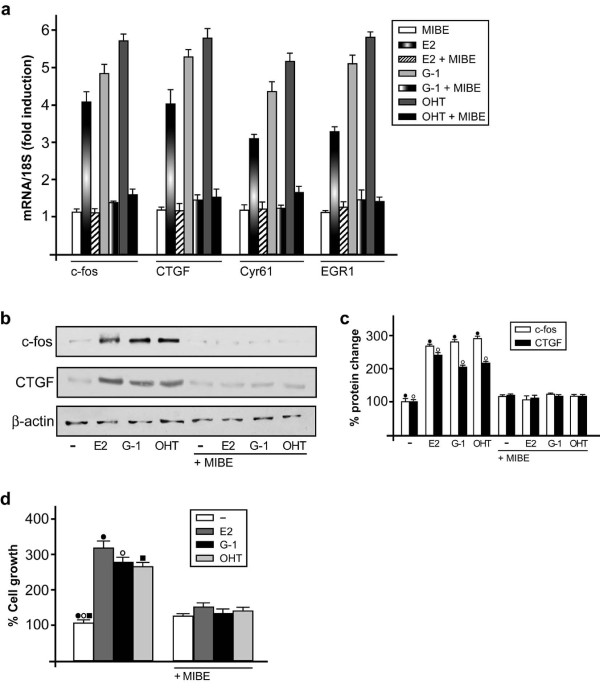
**MIBE inhibits GPER target genes and proliferation induced by E2, G-1 and OHT**. (a) The expression of c-fos, CTGF, Cyr61 and EGR1 induced in SkBr3 cells by 1 h treatment with 100 nM E2, 1 microM G-1 and 5 microM OHT is inhibited in presence of 10 microM MIBE, as evaluated by real-time PCR. Results obtained from experiments performed in triplicate were normalized for 18S expression and shown as fold change of RNA expression compared to cells treated with vehicle. Each data point represents the mean ± SD of three independent experiments performed in triplicate. (b) The up-regulation of c-fos and CTGF protein levels induced in SkBr3 cells by 2 h treatment with 100 nM E2, 1 microM G-1 and 5 microM OHT were abolished in presence of 10 microM MIBE. Data shown are representative of three independent experiments. beta-actin serves as a loading control. (c) Densitometric analysis of c-fos and CTGF protein expressions normalized to beta-actin. (•), (◦) indicate *P *< 0.05 for cells receiving vehicle versus treatments. (d) The proliferation of SkBr3 cells upon treatment with 100 nM E2, 100 nM G-1 and 100 nM OHT was inhibited by 1 microM MIBE, as indicated. Cells were treated for five days with the indicated treatments and counted on Day 6. Proliferation of cells receiving vehicle was set as 100% upon which cell growth induced by treatments was calculated. Each data point is the average ± SD of three independent experiments performed in triplicate. (•), (◦), (▪), indicate *P *< 0.05 for cells receiving vehicle (-) versus treatments.

The characterization of the transcriptional response to GPER signaling has recently identified a set of target genes that mediate the stimulatory effects triggered by GPER activation in cancer cells [24]. Hence, we performed real-time PCR experiments to evaluate the potential of MIBE in regulating the expression of GPER-dependent genes. Of note, the up-regulation of c-fos, CTGF, Cyr61 and EGR1 induced by the GPER agonists E2, G-1 and OHT in SKBr3 cells was abolished in the presence of MIBE (Figure 7a). In accordance with these results, MIBE also prevented the increase of both c-fos and CTGF at the protein level (Figure 7b, c). Next, we wondered what might be the biological significance of the inhibitory action of MIBE through GPER signaling. As shown in panel d of Figure 7, the proliferative effects elicited by E2, G-1 and OHT in SKBr3 cells were inhibited by MIBE. Altogether, these findings demonstrate that MIBE acts as an antagonist of both ERα and GPER in breast cancer cells.

## Discussion

In the present study, we identified the first ligand of ERα and GPER, referred to as MIBE, which acts as an antagonist of both receptors in breast cancer cells. By molecular modeling and binding experiments we demonstrated that MIBE binds to both receptors, while through functional assays we showed that MIBE inhibits the ERα- and GPER-mediated signaling. In particular, using the ER-positive MCF7 and ER-negative SkBr3 breast cancer cells as a model system, we characterized the biological properties of MIBE. We found that in MCF7 cells MIBE blocks the ER transactivation induced by E2 as well as the ER-mediated gene regulation and cell proliferation. In addition, in SkBr3 cells MIBE prevented the GPER-dependent responses, such as rapid ERK phosphorylation, gene transcription and growth effects induced by the GPER agonists E2, OHT and G-1. The exclusive antagonistic action exerted by MIBE on both ERα and GPER could represent a novel promising tool for a more comprehensive pharmacological approach in estrogen-dependent tumors like breast cancer, which express one or both receptors from the onset or following tumor progression.

Breast cancer is the most commonly diagnosed invasive malignancy and the second leading cause of cancer death in women [42]. Endocrine treatment along with surgery, chemotherapy, radiotherapy and targeted therapy are fundamental modalities for the therapeutic management of breast cancer. The expression of ERα in breast carcinomas correlates with the beneficial response to anti-estrogens [43], whereas the lacking of ERα is coupled to a worse prognosis and to short disease-free survival rates [44]. On the basis of the main role exerted by ERα in the development and progression of breast cancer and considering that this receptor is expressed in approximately 70% of breast tumors, the ER antagonist tamoxifen has been widely used, although its effectiveness is limited by *de novo *and acquired resistance [45]. In accordance with these data, comparative clinical studies have indicated that aromatase inhibitors blocking estrogen biosynthesis may provide major benefits in respect to ERα antagonists in breast cancer patients [46]. Among the various mechanisms involved in the resistance to endocrine treatment, the activation of transduction pathways different from those mediated by ERα has been proposed. For instance, an increased expression and/or activation of growth factor receptors, such as EGFR/HER2, have been associated with the failure of endocrine therapy in breast tumors [47]. Moreover, the existence of alternative ERs able to mediate estrogen signaling without exhibiting any sensitivity to the repressive action of the ER antagonists could be also involved in the resistance to endocrine agents. In this scenario, it has been recently demonstrated that GPER acts as an additional receptor mediating the effects of estrogens in a wide number of cell types, such as breast, endometrial and ovarian cancer cells [7]. Of note, diverse studies have shown that E2 as well as the anti-estrogens tamoxifen and ICI bind to and activate GPER signaling, including ERK phosphorylation and gene transcription, which in turn lead to cancer cell proliferation and migration [7].

The activation of the GPER transduction pathway requires the EGFR transactivation [8], in accordance with evidence showing that the agonist stimulation of diverse G-protein coupled receptors (GPCRs) triggers the transactivation of EGFR through the release of EGF-like ligands tethered at the cell surface and the subsequent generation of intracellular signaling [48]. In addition, the functional crosstalk which occurs between members of GPCR and growth factor receptor families contributes to the progression of different tumors [8,48]. In this regard, we have previously reported that GPER and EGFR physically and functionally interact in both ER-negative and ER-positive cancer cells [22,23]. Recently, it has also been found that a crosstalk among EGFR, the nerve growth factor (NGF) receptor TrkA and the GPCR Formyl Peptide Receptor (FPR) occurs in monocytes [49]. In particular, the inhibition of EGFR prevented the ligand-dependent responses mediated by the other two receptors, while the inhibition of FPR abolished the EGFR and TrkA phosphorylation induced by EGF and NGF, respectively. Accordingly, the silencing of each receptor suppressed the capability of the other receptors to mediate the ligand-induced actions like ERK phosphorylation [49]. In line with these findings, our current results provide novel insight into the functional crosstalk between GPER and EGFR in cancer cells. Notably, we show for the first time that the activation of EGFR induced by its cognate ligand EGF is abolished by knocking down GPER expression or in the presence of MIBE, which is an inhibitor of GPER as ascertained in the present study. Nevertheless, further studies are needed to better understand the role played by GPER in the activation of EGFR by its cognate ligand EGF and to appreciate the potential of MIBE in preventing the crosstalk between GPER and EGFR which was previously well described [23].

On the basis of these remarks, it remains to be evaluated that the potential of MIBE to interfere with the functional crosstalk between EGFR and ERα, toward a better characterization of its inhibitory activity elicited in cell contexts expressing both receptors. In particular, considering that a physical and functional interaction between EGFR and ER leads to the activation of multiple intracellular cascades, including MAPK, phosphoinositide 3-kinase (PI3K) and other protein kinases [50-53], it would be interesting to ascertain whether MIBE could alter these transduction signals that have been involved in the proliferation of cancer cells [50,54-58].

In 2005, two reports provided evidence on the capability of estrogens and anti-estrogens to bind to GPER [10,19]. In particular, the ER antagonists tamoxifen and ICI displayed a high binding affinity for GPER, as assessed in competition assays. Surprisingly, unlike the antagonistic properties exhibited by these agents on the classical ER-mediated pathways, both tamoxifen and ICI act as GPER agonists [8,9,19]. In the following years, further ER ligands and activators showed the ability to bind to GPER eliciting promiscuous actions through the two receptors. For instance, the phytoestrogen genistein and the xenoestrogen bisphenol A, which exert estrogen-like activities binding to and activating ERα [9,59], displayed the ability to bind to and activate GPER signaling [9,27,60]. As it concerns the pesticide atrazine, it exerted estrogenic effects without binding to ERs [61] and exhibiting the capability to activate the GPER-mediated pathway despite a low binding affinity for this receptor [25,27]. Unlike E2 which exhibited ERα and GPER agonism in several investigations [7], the well known ERα ligand and activator estriol showed antagonistic properties for GPER-mediated signaling [28]. Besides, G-1 [29] and G-15, along with its derivatives [30,31] as ligands activated or inhibited, respectively, the GPER-mediated signaling, while some GPER antagonists triggered at high concentrations ER-dependent transcriptional responses [30].

GPER expression was indicated as a potential predictor of biological aggressive features in breast carcinomas [16]. Although a significant association between ERα and GPER was observed, approximately 50% of ERα-negative breast tumors retained GPER suggesting that the expression of these receptors may not be interdependent [16]. On the basis of these and the aforementioned findings, tumor cells that express GPER but lack ERα may be stimulated by estrogens and even by anti-estrogens, such as tamoxifen. In this regard, it should be noted that the stimulatory effects on cancer progression elicited by estrogens via both ERα and GPER and by ERα antagonists through GPER address the need to discover novel drugs targeting simultaneously both receptors, in order to obtain major therapeutic benefits in respect to the use of the current selective antagonists.

## Conclusions

**Figure 8:**
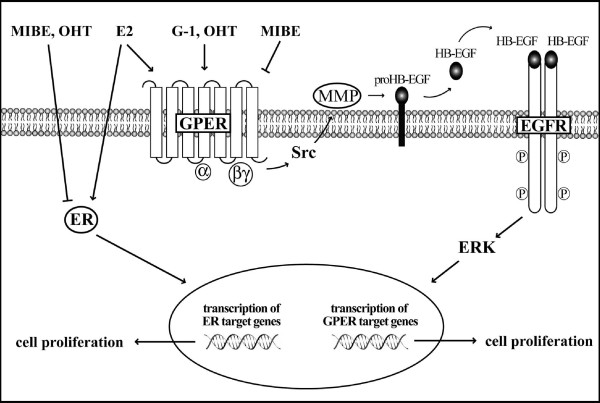
**Schematic representation of the inhibitory activity exerted by MIBE on GPER- and ER-mediated signaling**.

The exclusive antagonistic activity exerted by MIBE on ERα- and GPER-mediated signaling as shown in the present study (Figure 8), could represent a promising pharmacological approach either at the beginning or during the progression of breast tumors which express one or both receptors. In this respect, further studies are needed to examine whether MIBE could be considered a useful tool towards a more comprehensive treatment in breast cancer.

## Abbreviations

AR: androgen receptor; CAFs: cancer associated fibroblasts; CS: charcoal-stripped; DBD: DNA binding domain; DHT: 5α-dihydrotestosterone; DMSO: dimethyl sulfoxide; E2: 17β-estradiol; EGFR: epidermal growth factor receptor; ER: estrogen receptor; ERK: extracellular signal-regulated kinase; FPR: formyl peptide receptor; G-1: 1-[4-(6-bromobenzo[1:3]dioxol-5-yl)-3a:4:5:9b-tetrahydro-3H-cyclopenta[c]quinolin-8-yl]-ethanone; G-15: 4-(6-Bromobenzo[1:3]dioxol-5-yl)-3a:4:5:9b-tetrahydro-3H-cyclopenta[c]quinoline; GPCRs: G-protein coupled receptors; GPER: G protein-coupled estrogen receptor; HB-EGF: heparan-bound epidermal growth factor; LBD: ligand binding domain; MAPK: mitogen-activated protein kinase; MIBE: ethyl 3-[5-(2-ethoxycarbonyl-1-methylvinyloxy)-1-methyl-1H-indol-3-yl]but-2-enoate; NGF: nerve growth factor; OHT: 4-hydroxytamoxifen; PI3K: phophatidylinositol 3-kninase; PLC: phospholipase C; SDS: sodium dodecyl sulfate; TLC: thin layer chromatography.

## Competing interests

The authors declare that they have no competing interests.

## Authors' contributions

RL designed and performed the experiments, and wrote the paper. MFS and MP performed the experiments. MSS and AC synthesized MIBE. CR performed docking simulations. MM analyzed data and wrote the paper. All authors read and approved the final manuscript for publication.

## Supplementary Material

Additional file 1 **MIBE does not activate AR**. Hek293 cells were transfected with AR luciferase reporter gene (ARE-luc) and AR expression plasmid along with the internal transfection control Renilla Luciferase, and treated with 10 nM DHT alone and in combination with 10 μM MIBE, as indicated. The normalized luciferase activities of cells treated with vehicle (-) were set as one-fold induction, upon which the activities induced by treatments were calculated. Each data point represents the mean ± SD of three experiments performed in triplicate. Click here for file

Additional file 2 **MIBE prevents the phosphorylation of EGFR induced by G-1**. **(a) **EGFR^Tyr1173 ^phosphorylation after treatment (30 minutes) with vehicle (-) and 1 μM G-1 alone and in combination with 10 μM MIBE. **(b) **Densitometric analysis of three independent experiments, EGFR^Tyr1173 ^expressions are normalized to EGFR. Click here for file
