# Supplementary figures and images for: MIBE acts as antagonist ligand of both estrogen receptor α and GPER in breast cancer cells
Source: Breast Cancer Res. 2012 Jan 17;14(1):R12. doi: 10.1186/bcr3096 (PMC3496129; doi:10.1186/bcr3096)

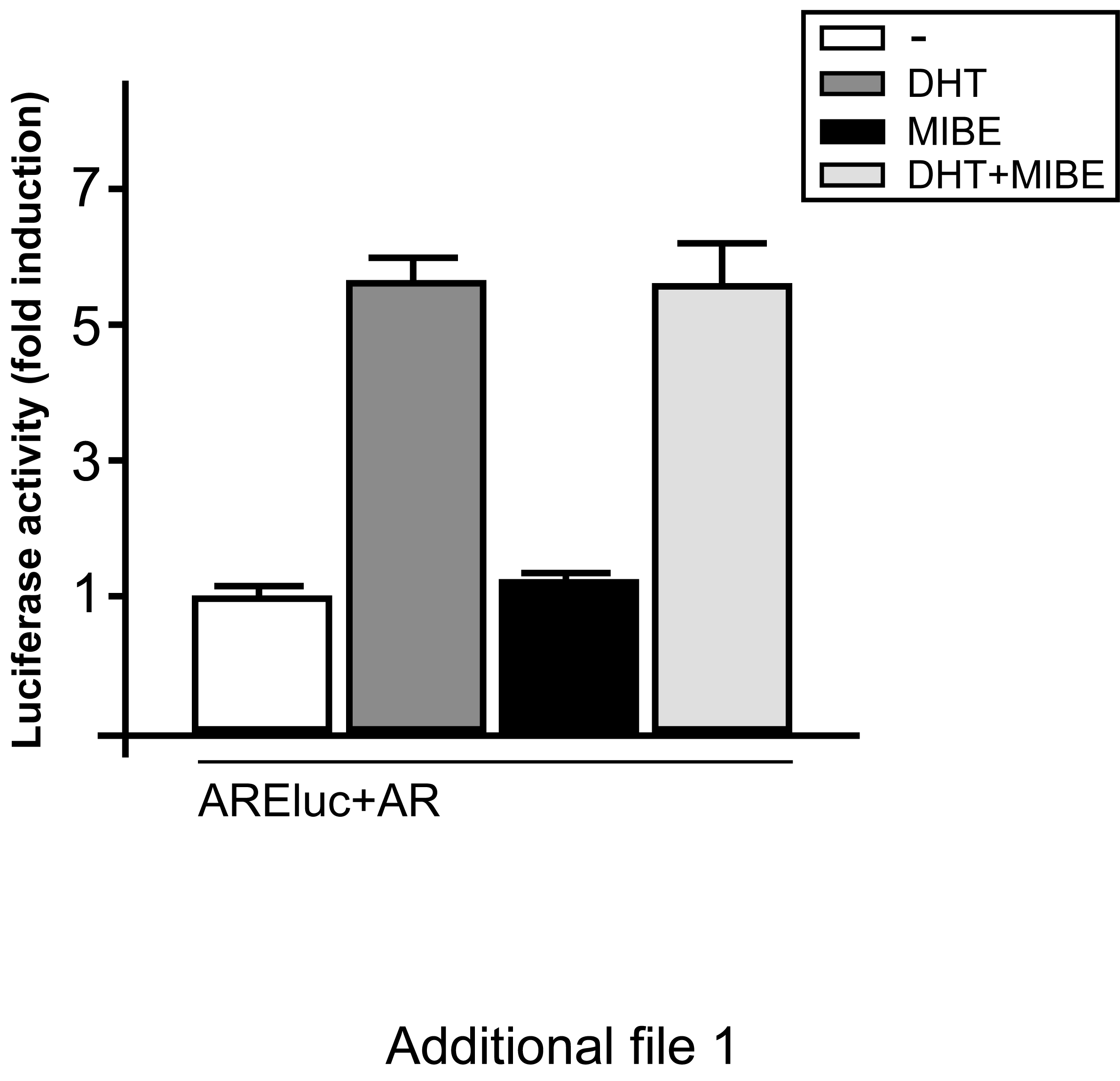

Supplement: Additional file 1 — MIBE does not activate AR. Hek293 cells were transfected with AR luciferase reporter gene (ARE-luc) and AR expression plasmid along with the internal transfection control Renilla Luciferase, and treated with 10 nM DHT alone and in combination with 10 μM MIBE, as indicated. The normalized luciferase activities of cells treated with vehicle (-) were set as one-fold induction, upon which the activities induced by treatments were calculated. Each data point represents the mean ± SD of three experiments performed in triplicate. [file bcr3096-S1.TIFF]

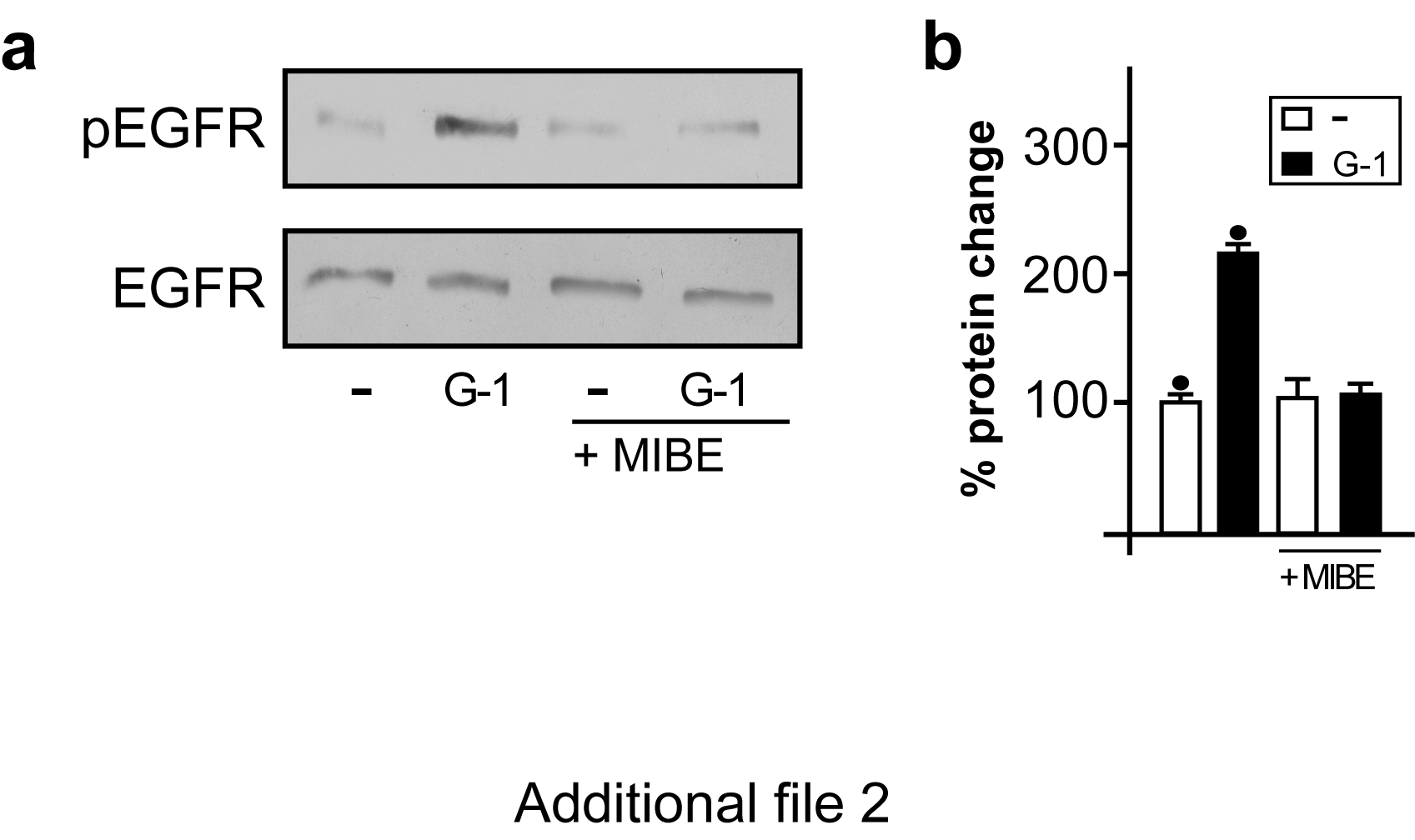

Supplement: Additional file 2 — MIBE prevents the phosphorylation of EGFR induced by G-1. (a) EGFRTyr1173 phosphorylation after treatment (30 minutes) with vehicle (-) and 1 μM G-1 alone and in combination with 10 μM MIBE. (b) Densitometric analysis of three independent experiments, EGFRTyr1173 expressions are normalized to EGFR. [file bcr3096-S2.TIFF]
